# Outcomes of right-sided and left-sided colon cancer after curative resection

**DOI:** 10.1038/s41598-022-15571-2

**Published:** 2022-07-05

**Authors:** Chien-Yi Yang, Min-Hsuan Yen, Kee-Thai Kiu, Yu-Ting Chen, Tung-Cheng Chang

**Affiliations:** 1 Department of Surgery, Taipei Medical University Shuang-Ho Hospital, No. 291, Zhongzheng Road, Zhonghe District, New Taipei City, 235 Taiwan; 2 Division of Colorectal Surgery, Department of Surgery, Taipei Medical University Shuang-Ho Hospital, No. 291, Zhongzheng Road, Zhonghe District, New Taipei City, 235 Taiwan; 3 Department of Surgery, School of Medicine, College of Medicine, Taipei Medical University, New Taipei City, Taiwan

**Keywords:** Cancer, Gastroenterology, Oncology, Risk factors

## Abstract

The right and left side of the colon derived from the midgut and hindgut, respectively. Previous studies have reported different characteristics of right-sided colon cancer (RCC) and left-sided colon cancer (LCC), but oncological outcomes remain unclear. This study compared the outcomes of RCC and LCC. This retrospective study included 1017 patients who received curative colectomy for stage I-III colon cancer at a single institute between August 2008 and December 2019. Overall survival (OS) and time to recurrence (TTR) were analyzed as outcome measurements. No significant difference in the OS or TTR of patients with RCC and LCC were observed. In subgroup analysis, RCC was associated with shorter TTR than LCC in stage II colon cancer (HR 2.36, 95% confidence interval 1.24–4.48, *p* < 0.01). Multivariate analysis demonstrated that right sidedness, R1 resection, low body mass index (BMI) and adjuvant chemotherapy were independent factors for poor prognosis for stage II colon cancer. Low BMI, perineural invasion, higher T stage and N2 stage were independent factors for poor prognosis for stage III colon cancer. The results were confirmed by multivariate analysis after propensity score matching. Our study revealed that RCC was an independent risk factor for recurrence in stage II colon cancer.

## Introduction

Colorectal cancer is one of the most prevalent cancers, especially in developed countries^1,2^. Radical surgical resection is the standard treatment for American Joint Committee on Cancer (AJCC) stage I to III colon cancer; postoperative adjuvant chemotherapy is also administered to patients with high-risk stage II and stage III colon cancer^3^.

Since 1990, right-sided colon cancer (RCC) and left-sided colon cancer (LCC) have been regarded as distinct cancers based on their different embryology, epidemiology, pathology, and prognosis^4,5^. Patients with RCC are more likely to be older and female and present with a more advanced tumor stage, larger tumor size, and more poorly differentiated tumor cells than those with LCC^6–11^. The differing characteristics of RCC and LCC are thought to be caused by differences in embryologic origin, fecal exposure, and detection time^6^. Most previous studies have indicated that RCC was associated with a higher recurrence and lower survival rate than LCC^6,12,13^, although several studies have concluded that early-stage RCC had a better prognosis than LCC^14,15^. In light of the lack of consensus on the prognosis for RCC and LCC, this study investigated the impact of cancer sidedness and stage on outcomes.

## Material and methods

### Patients

**Figure 1:**
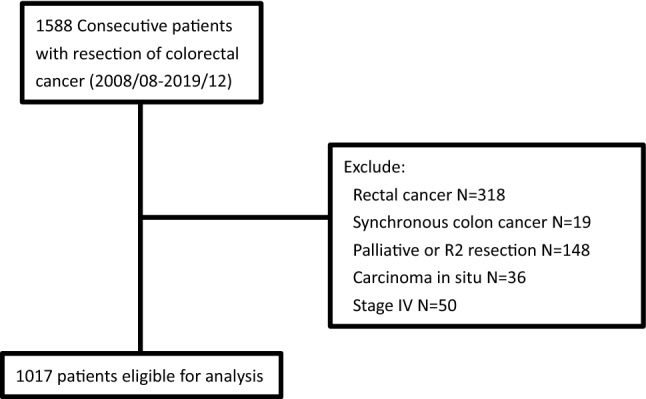
Flow diagram of colorectal cancer patients included in the study.

The medical records of 1588 consecutive patients who received primary resection of colorectal cancer in Taipei Medical University Shuang-Ho Hospital between August 2008 and December 2019 were reviewed. The TNM staging system (*AJCC Cancer Staging Manual, 8th Edition*) was used for staging. Only patients with colon adenocarcinoma were included in this study. Patients who were diagnosed with rectal cancer, carcinoma in situ, synchronous colon cancer, or stage IV colon cancer were excluded, as were patients who received palliative surgery or R2 resection. The final sample comprised 1017 patients who underwent curative resection for stage I, II, or III colon cancer (Fig. 1). Metachronous cancers were defined as different individuals with different diseases instead of same patients with cancer recurrence. This study was approved by the Institutional Review Board/Ethics Committee of Taipei Medical University (approval number: N201912114). As this is a retrospective study, the informed consent is not required by TMU-JIRB.

### Outcomes measurement

Primary tumors located at the cecum, ascending colon, and transverse colon were defined as RCC, whereas primary tumors located at the splenic flexure, descending colon, and sigmoid colon were defined as LCC. Patient characteristics including age at diagnosis, gender, medical history, body mass index (BMI), carcinoembryonic antigen (CEA) value, tumor location, histological type, tumor grade, tumor stage, chemotherapy history, K-RAS status, mismatch repair (MMR) status and surgical margin status were collected from patient records. R1 resection was defined as section margin less than 2 mm. Overall survival (OS) was defined as the time from primary tumor resection to death from any cause. Time to recurrence (TTR) was defined as the time from primary tumor resection to the first recurrence confirmed by radiological or histological features.

### Statistical analysis

Both K–S and S–W tests were used to confirmed the normal distribution of continuous variables. Variables normally distributed were presented as mean ± standard deviation, otherwise presented as median (Q1–Q3). Comparisons were made using independent-t test or Mann–Whitney test for analysis of continuous variables. Chi-square test was used for comparisons of categorical variables. Kaplan–Meier curves were calculated for all patients and patients with stage I, II, and III colon cancer separately to compare OS and TTR between patients with RCC and LCC. Univariate and multivariate survival analyses were performed with a Cox proportional hazards function; HRs and 95% confidence intervals (CIs) were used to estimate the impact of primary tumor location on survival outcomes. Propensity score matching (PSM) was performed with greedy nearest neighbor matching method, using 14 variables (age, sex, BMI, patient origin, operation method, CEA, LVI, PNI, tumor grade, T stage, N stage, tumor size, chemotherapy and R1 resection) that could potentially influence outcomes. The number of lymph node harvested was not included due to native difference of mesocolon taken during operation between RCC and LCC. The caliper requirement was ignored. A *p* value of < 0.05 was considered statistically significant.

### Ethics approval

This study was approved by the Ethics Committee of Taipei Medical University (approval number: N201912114) and was performed in accordance with the Declaration of Helsinki and its subsequent amendments or comparable ethical standards.

### Informed consent

Informed consent was not applicable due to the retrospective nature of this study.

## Results

### Clinical and pathological characteristics

We reviewed the medical records of 1588 patients, and 1017 patients were eligible for inclusion in the study. 75% of the patients were followed for more than 48 months. Ten patients were found metachronous cancers. Of the included patients, 385 (37.9%) had RCC, and 632 (62.1%) had LCC (Fig. 1). The baseline characteristics of patients with RCC and LCC are listed in Table 1. Patients with RCC were older than patients with LCC (67 (59–77) years vs. 65.3 (57–75) years, *p* < 0.01) and less likely to be male (47.3% vs. 56.5%, *p* < 0.01). A lower BMI was also noted in patients with RCC than in patients with LCC (23.7 ± 4.4 vs. 24.5 ± 4.1, *p* < 0.01). Minimally invasive surgeries were performed more frequently in patients with LCC than in patients with RCC (62.3% vs. 47.5%, *p* < 0.01). No significant differences in preoperative CEA level, the rate of emergency operations, the rate of adjuvant chemotherapy, T stage, or postoperative follow-up time were observed. More lymph nodes were harvested in patients with RCC than in patients with LCC (26 (19–35.5) vs. 21 (16–28), *p* < 0.01). Tumors in patients with RCC were larger (4.5 (3.15–6.4) vs. 4.0 (3.0–5.5), *p* < 0.01) and of a more advanced histological grade (7.8% vs. 4.4%, *p* = 0.03) than the tumors in patients with LCC. However, patients with LCC had a more advanced N stage (53.6% vs. 43.6% in N1 and N2 stage, *p* = 0.01) and AJCC cancer stage (53.6% vs. 43.6% in stage III, *p* < 0.01) than did patients with RCC. A higher percentage of K-RAS mutation (48.5% vs. 31.6%, *p* < 0.01) and deficient MMR (dMMR) (45.5% vs. 18.2%, *p* < 0.01) were noted in patients with RCC than in patients with LCC. No significant differences in the rate of perineural invasion or lymphovascular invasion were apparent between the two groups. R1 resection was found in 17 with RCCand 19 patients with LCC with RCC and LCC. There is no significant difference between the two groups (4.4% vs. 3.0%, *p* = 0.24).

**Table 1:** Baseline characteristics of all patients.

Characteristic	Right-sided cancer (N = 385)	Left-sided cancer (N = 632)	*P* value
Age (years)	67(59–77)	65.3 (57–75)	** < 0.01**
Sex, Male, N (%)	182 (47.3%)	357 (56.5%)	** < 0.01**
Body mass index	23.7 ± 4.4	24.5 ± 4.1	** < 0.01**
**Patient origin, N (%)**			0.12
Elective operation	301 (78.2%)	519 (82.1%)	
Emergency operation	84 (21.8%)	113 (17.9%)	
**Operation method, N (%)**			** < 0.01**
Open surgery	202 (52.5%)	238 (37.7%)	
Minimal invasive surgery	183 (47.5%)	394 (62.3%)	
CEA	3.3 (1.7–9.4)	3.6 (1.9–8.7)	0.39
Tumor size (cm)	4.5 (3.2–6.4)	4.0 (3.0–5.5)	** < 0.01**
Lymphovascular invasion, N (%)	185 (48.1%)	304 (48.1%)	0.99
Perineural invasion, N (%)	148 (38.4%)	230 (36.4%)	0.51
**Tumor grade, N (%)**			**0.03**
Grade 1–2	355 (92.2%)	604 (95.6%)	
Grade 3–4	30 (7.8%)	28 (4.4%)	
**Location**			
Cecum, N (%)	70		
Ascending colon, N (%)	166		
Hepatic flexure, N (%)	50		
Transverse colon, N (%)	99		
Splenic flexure, N (%)		35	
Descending colon, N (%)		38	
Descending-sigmoid junction, N (%)		46	
Sigmoid colon, N (%)		513	
**T stage, N (%)**			
T1	41 (10.6%)	86 (13.6%)	0.05
T2	39 (10.1%)	83 (13.1%)	
T3	214 (55.6%)	351 (55.5%)	
T4	91 (23.6%)	112 (17.7%)	
**N stage, N (%)**			
N0	217 (56.4%)	293 (46.4%)	**0.01**
N1	106 (27.5%)	221 (35.0%)	
N2	62 (16.1%)	118 (18.7%)	
**AJCC stage, N (%)**			
I	66 (17.1%)	122 (19.3%)	** < 0.01**
II	151 (39.2%)	171 (27.1%)	
III	168 (43.6%)	339 (53.6%)	
No. of lymph node harvested	26 (19–35.5)	21 (16–28)	** < 0.01**
Adjuvant chemotherapy, N (%)	197 (51.2%)	323 (51.1%)	0.99
K-ras mutation, No. of positive/No. of test (%)	94/194 (48.5%)	93/294 (31.6%)	** < 0.01**
dMMR, No. of positive/No. of test (%)	25/55 (45.5%)	10/55 (18.2%)	** < 0.01**
MSI-H, No. of positive/No. of test (%)	5/20 (25%)	1/13 (7.7%)	0.21
R1 resection, N (%)	17 (4.4%)	19 (3.0%)	0.24
Follow time (month)	37.8 (23.5–63.5)	43.0 (25.3–67.5)	0.12

Significant values are in bold.

*CEA* carcinoembryonic antigen, *dMMR* Deficient mismatch repair, *MSI-H* microsatellite instability-high.

### Clinical and pathological characteristics stratified by stage

The study included 188, 322, and 507 patients with stage I, II, and III colon cancer, respectively. Table 2 presents the differences in characteristics between each stage. Men made up a smaller proportion of the patients with stage I and II RCC than that of the patients with stage I and II LCC (stage I: 42.4% vs. 58.2%, *p* = 0.04; stage II: 45% vs. 56%, *p* = 0.047). Patients with stage III LCC had a higher BMI than patients with stage III RCC (23.4 ± 4.6 vs. 24.2 ± 3.8, *p* = 0.04). More patients with stage II and III LCC underwent minimally invasive surgery than did patients with stage II and III RCC (stage II: 56.1% vs. 43.0%, *p* = 0.02; stage III: 61.1% vs. 43.5%, *p* < 0.01). Lymphovascular invasion was higher in patients with stage II RCC than in patients with stage II LCC (40.4% vs. 28.7%, *p* = 0.03). Tumors in stage I and II RCC had more advanced histological grading than did tumors in stage I and II LCC (stage I: 6.1% vs. 0.8%, *p* = 0.03; stage II: 11.9% vs. 4.1%, *p* = 0.01). A higher rate of K-RAS mutation and dMMR was noted in stage II RCC than in stage II LCC (K-RAS: 44.2% vs. 28.9%, *p* = 0.04; dMMR: 46.2% vs. 15%, *p* = 0.03) and in stage III RCC than in stage III LCC (K-RAS: 53.2% vs. 31.2%, *p* < 0.01; dMMR: 47.6% vs. 10.7%, *p* < 0.01). More lymph nodes were harvested in RCC than LCC in all three stages (stage I: 22 (18.75–31) vs. 17 (14–24), *p* < 0.01; stage II: 24 (18–35) vs. 22 (17–29), *p* = 0.02; and stage III: 29 (21–38.75) vs. 22 (17–30), *p* < 0.01). More R1 resection was found in stage III RCC than in stage III LCC (7.7% vs. 3.5%, *p* = 0.04).

**Table 2:** Comparison of clinicopathological characteristics between right-sided and left-sided in stage I, II and III colon cancer.

Characteristic	Stage I			Stage II			Stage III		
	Right-sided cancer (N = 66)	Left-sided cancer (N = 122)	*P* value	Right-sided cancer (N = 151)	Left-sided cancer (N = 171)	*P* value	Right-sided cancer (N = 168)	Left-sided cancer (N = 339)	*P* value
Age (years)	64.5 (57–74)	62 (55.8–67.3)	0.16	70 (59–79)	66 (58–77)	0.12	68 (60–77.75)	65 (57–76)	0.11
Sex, Male, N (%)	28 (42.4%)	71 (58.2%)	**0.04**	68 (45%)	96 (56%)	**0.047**	86 (51.1%)	190 (56.0%)	0.30
Body mass index	24.9 ± 3.7	25.9 ± 4.4	0.44	23.6 ± 4.3	24.2 ± 4.3	0.25	23.4 ± 4.6	24.2 ± 3.8	**0.04**
**Patient origin, N (%)**			0.74			0.13			0.65
Elective operation	62 (93.9%)	116 (95.1%)		111 (73.5%)	138 (80.7%)		128(76.1%)	265(78.2%)	
Emergency operation	4 (6.1%)	6 (4.9%)		40 (26.5%)	33 (19.3%)		40(23.9%)	74(21.8%)	
**Operation method, N (%)**			0.35			**0.02**			** < 0.01**
Open surgery	21 (31.8%)	31 (25.4%)		86 (57.0%)	75 (43.9%)		95 (56.5)%	132 (38.9%)	
Minimal invasive surgery	45 (68.2%)	91 (74.6%)		65 (43.0%)	96 (56.1%)		73 (43.5%)	207 (61.1%)	
CEA	2.1 (1.4–3.3)	2.0 (1.4–3.0)	0.80	3.8 (1.9–10.1)	4.4 (2.1–10.2)	0.54	4.28 (2.0–13.4)	4.3 (2.2–11.3)	0.54
Tumor size (cm)	2.3 (1.5–3.7)	2.4 (1.5–3.5)	0.97	5.0 (3.5–7.0)	4.5 (3.5–6.0)	0.19	5.0 (4.0–6.5)	4.2 (3.2–5.5)	** < 0.01**
Lymphovascular invasion, N (%)	8 (12.1%)	23 (18.9%)	0.24	61 (40.4%)	49 (28.7%)	**0.03**	116 (69.0%)	232 (68.4)	0.89
Perineural invasion, N (%)	8 (12.1%)	6 (4.9%)	0.07	48 (31.8%)	50 (29.2%)	0.62	92 (54.8%)	174 (51.3%)	0.47
**Tumor grade, N (%)**			**0.03**			**0.01**			0.48
Grade 1–2	62 (93.9%)	121 (99.2%)		133 (88.1%)	164 (95.9%)		157 (93.5%)	322 (95.0%)	
Grade 3–4	4 (6.1%)	1 (0.8%)		18 (11.9%)	7 (4.1%)		11 (6.5%)	17 (5.0%)	
**T stage, N (%)**			0.57			0.13			**0.02**
T1	35 (53.0%)	70 (57.3%)					6 (3.6%)	16 (4.7%)	
T2	31 (47.0%)	52 (42.7%)					8 (4.8%)	31 (9.1%)	
T3				118 (78.1%)	145 (84.8%)		96 (57.1%)	206 (60.8%)	
T4				33 (21.9%)	26 (15.2%)		58 (34.5%)	86 (25.4%)	
**N stage, N (%)**									0.64
N0	66	122		151	171				
N1							106 (63.1%)	221 (65.2%)	
N2							62 (36.9%)	118 (34.8%)	
No. of lymph node harvested	22 (18.8–31)	17 (14–24)	** < 0.01**	24 (18–35)	22 (17–29)	**0.02**	29 (21–38.8)	22 (17–30)	** < 0.01**
Adjuvant chemotherapy, N (%)	1 (1.5%)	0 (0%)	0.18	72 (47.7%)	75 (43.9%)	0.49	124 (73.8%)	248 (73.2%)	0.88
K-ras mutation, no. of positive/No. of test (%)	10/23 (43.5%)	13/32 (40.6)	0.84	34/77 (44.2%)	22/76 (28.9%)	**0.04**	50/94 (53.2%)	58/186 (31.2%)	** < 0.01**
dMMR, no. of positive/no. of test (%)	3/8 (37.5%)	4/7 (57.1%)	0.45	12/26 (46.2%)	3/20 (15%)	**0.03**	10/21 (47.6%)	3/28 (10.7%)	** < 0.01**
MSI-H, no. of positive/no. of test (%)	0/1	0/3		4/10 (40%)	1/7 (14.3)	0.25	1/9 (11.1)	0/3	0.55
R1 resection, N (%)	0	1 (0.8%)	0.46	4 (2.6%)	6 (3.5%)	0.66	13 (7.7%)	12 (3.5%)	**0.04**
Follow time (month)	43.3 (29.5–67.4)	51.8 (30.1–78.1)	0.34	42.6 (15.6–66.0)	45.6 (28.5–73.3)	0.43	35.3 (19.5–58.5)	38.6 (22.0–61.1)	0.12

Significant values are in bold.

*CEA* carcinoembryonic antigen, *dMMR* Deficient mismatch repair, *MSI-H* microsatellite instability-high.

### Survival curves by tumor location and stage

**Figure 2:**
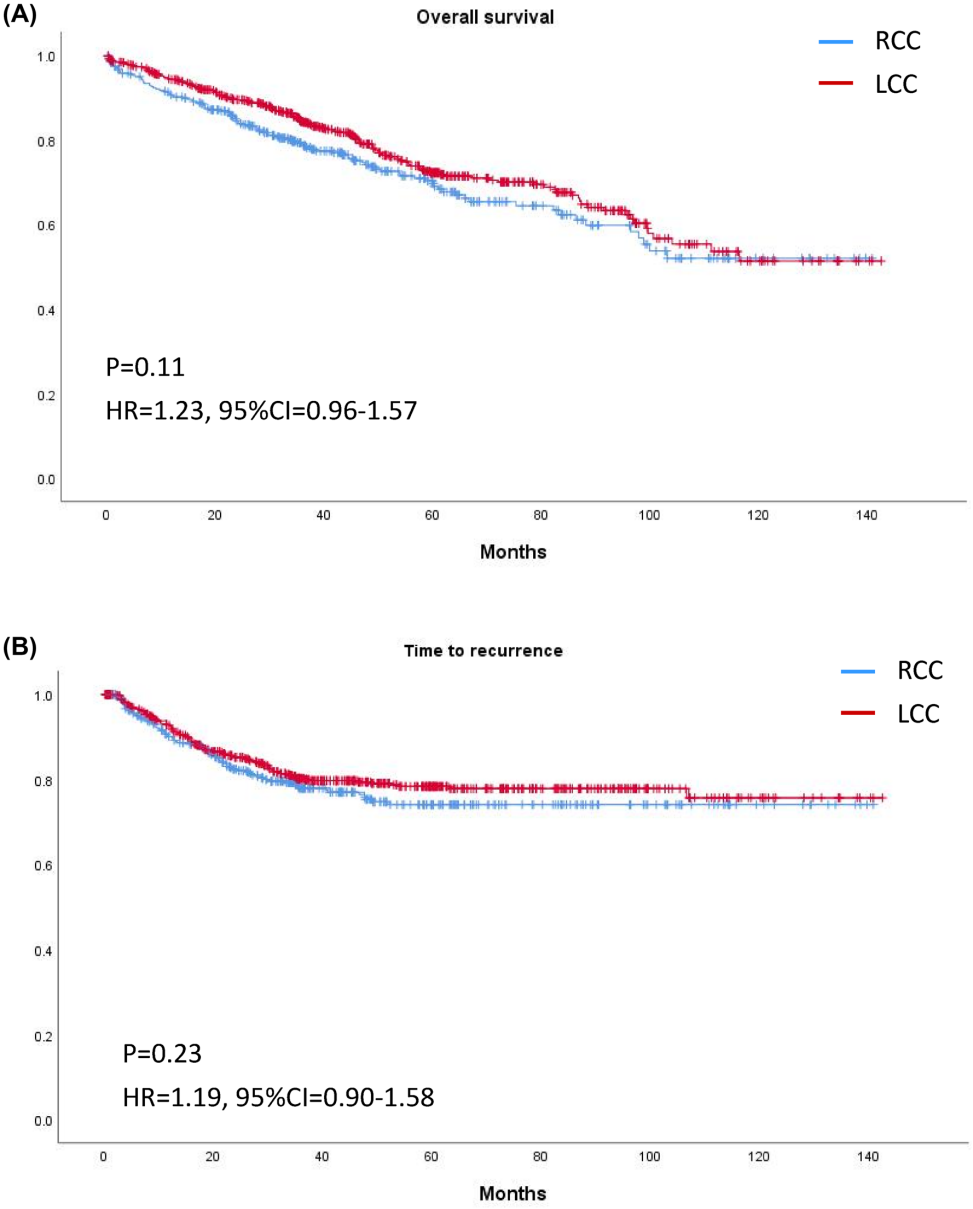
Kaplan–Meier curves of (**A**) overall survival and (**B**) time to recurrence of right-sided and left-sided colon cancer of any stage.

**Figure 3:**
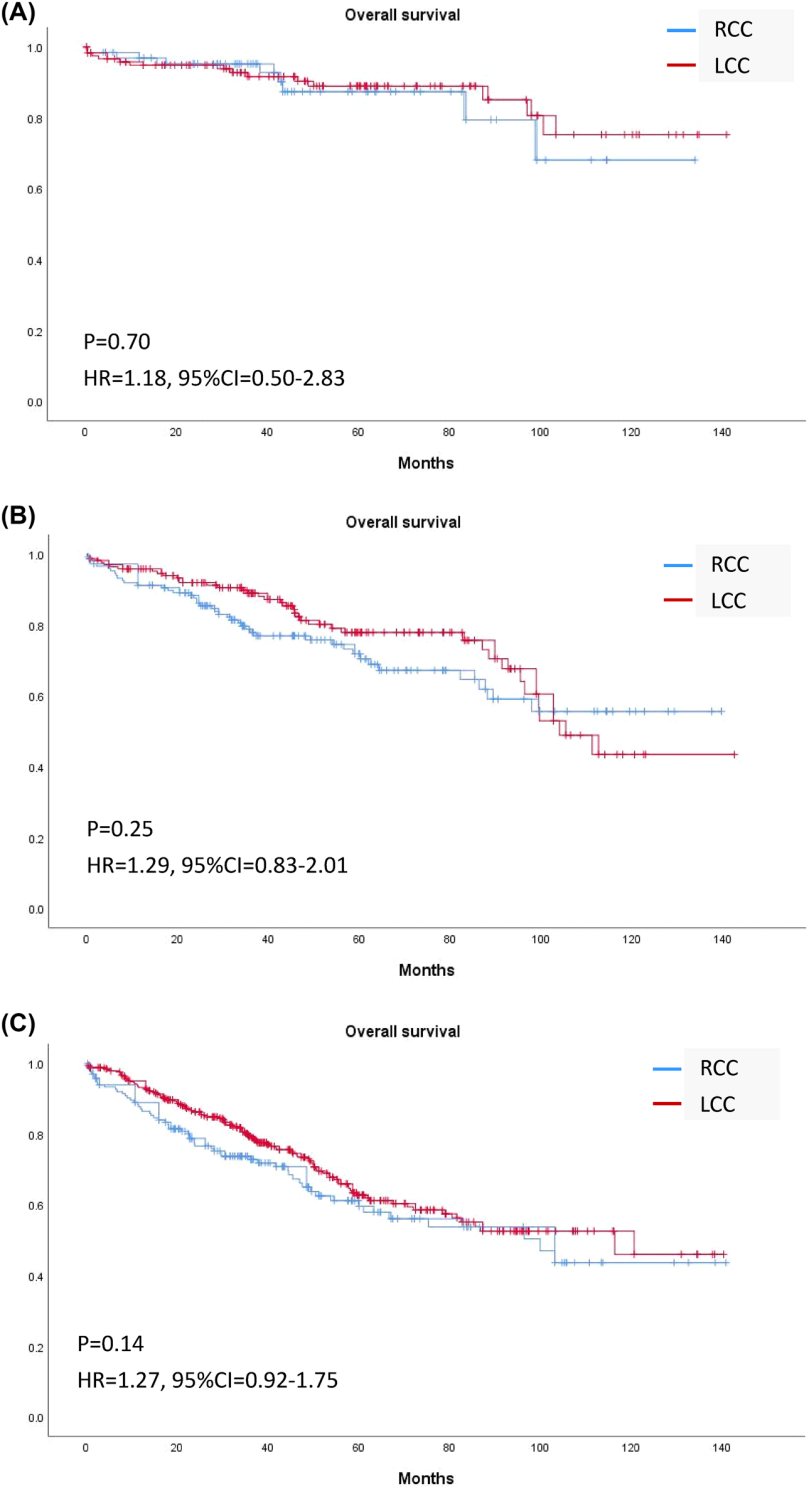
Kaplan–Meier curves of overall survival of right-sided and left-sided colon cancer in (**A**) Stage I, (**B**) Stage II, and (**C**) Stage III.

**Figure 4:**
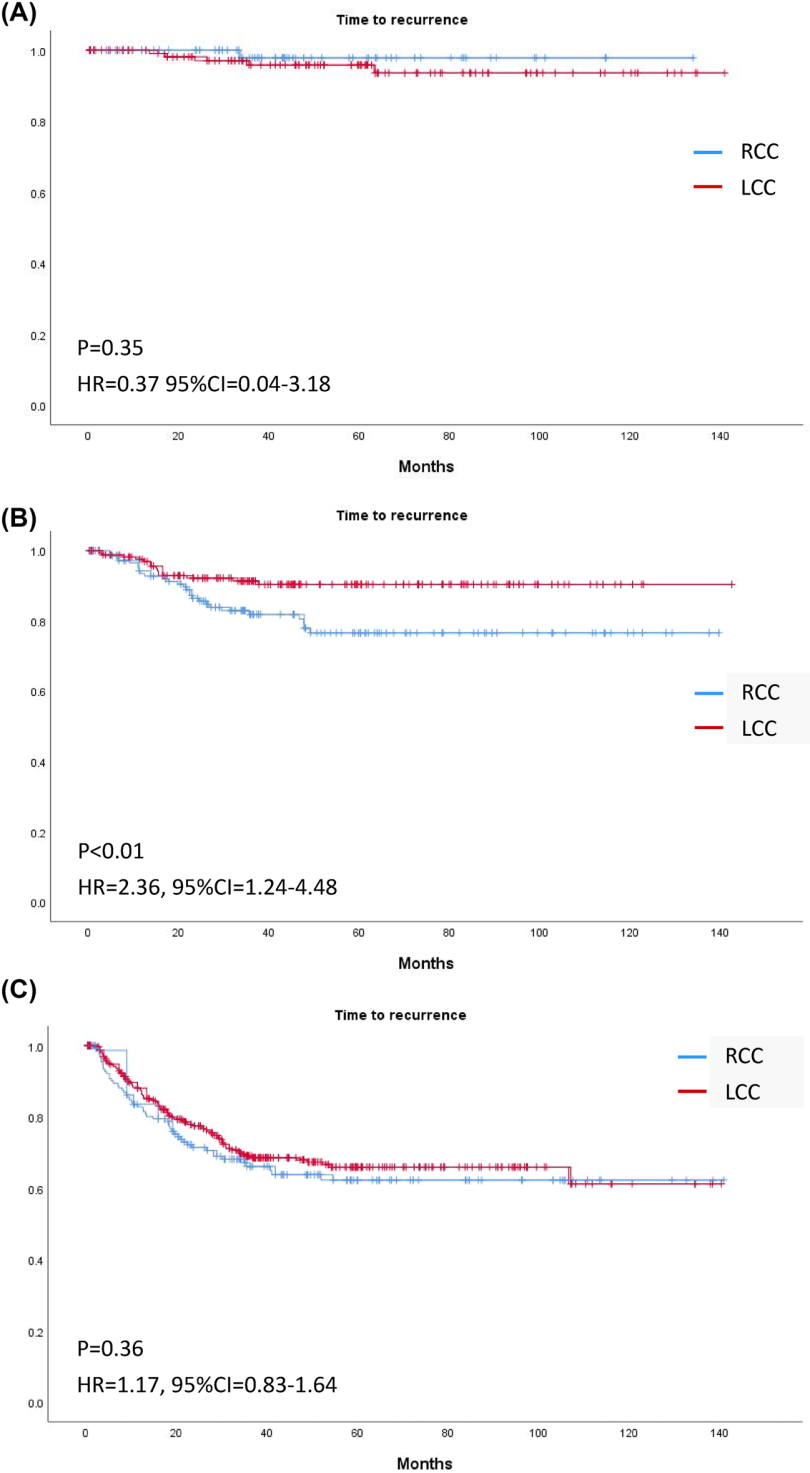
Kaplan–Meier curves of time to recurrence of right-sided and left-sided colon cancer in (**A**) Stage I, (**B**) Stage II, and (**C**) Stage III.

The median follow-up time was 37.8 (23.5–63.5) months in RCC and 43.0 (25.3–67.5) months in LCC (*p* = 0.12). Overall, Kaplan–Meier curves revealed no significant differences in OS or TTR for patients with RCC or LCC (Fig. 2). Stratification by stage revealed no significant difference in OS in RCC and LCC of any stage (Fig. 3); however, TTR in stage II RCC was shorter than that in stage II LCC (HR 2.36, 95% CI 1.24–4.48, *p* < 0.01; Fig. 4).

### Recurrent risks analysis in stage II and III colon cancer

In Table 3, we demonstrated recurrent risks (shorter TTR) by univariate and multivariate analysis. In univariate analysis, stage II RCC was associated with shorter TTR than was stage II LCC (HR 2.36, CI 1.24–4.48, *p* < 0.01). The risk factors for shorter TTR in stage II colon cancer were a higher BMI (HR: 0.88 for every increment in BMI, CI 0.81–0.97, *p* = 0.01), lymphovascular invasion (HR 2.19, CI 1.20–4.01, *p* = 0.01), perineurial invasion (HR 1.99, CI 1.08–3.66, *p* = 0.03), advanced T stage (HR 2.85, CI 1.52–5.36, *p* < 0.01), adjuvant chemotherapy (HR 2.28, CI 1.20–4.33, *p* = 0.01), and R1 resection (HR 6.35, CI 2.49–16.21, *p* < 0.01). The risk factors for shorter TTR in stage III colon cancer were higher BMI (HR 0.92 for every increment in BMI, CI 0.88–0.96, *p* < 0.01), older age (HR 1.02, CI 1.01–1.03, *p* = 0.01), emergency surgery (HR 2.36, CI 1.67–3.33, *p* < 0.01), open surgery (HR 1.39, CI 1.01–1.92, *p* = 0.046), perineural invasion (HR 2.15, CI 1.53–3.02, *p* < 0.01), advanced T stage (T2 vs T1 HR: 1.12, CI 0.1–12.33; T3 vs T1 HR 6.50 CI 0.9–46.96; T4 vs T1 HR 15.56 CI 2.16–112.2, *p* < 0.01), and N stage (HR 2.18, CI 1.59–3.02, *p* < 0.01; Table 3).

**Table 3:** Univariate and multivariate analysis^a^ for TTR by Cox Proportional Hazards Regression in stage II and stage III colon cancer.

Characteristic	Unadjusted analysis				Adjusted analysis			
	Stage II		Stage III		Stage II		Stage III	
	HR (95%CI)	*P* value	HR (95%CI)	*P* value	HR (95%CI)	*P* value	HR (95%CI)	*P* value
**Location**								
Right	2.36 (1.24–4.48)	** < 0.01** ^a^	1.17 (0.83–1.64)	0.36	2.35 (1.14–4.85)	**0.02**		
Left	1		1		1		1	
Age (for every 1 additional year in age)	0.99 (0.97–1.01)	0.45	1.02 (1.01–1.03)	**0.01**^a^			1.02 (0.99–1.03)	0.07
**Gender**								
Male	0.82 (0.45–1.51)	0.52	0.999 (0.72–1.38)	0.99				
Female	1		1				1	
BMI (for every 1 additional in BMI)	0.88 (0.81–0.97)	**0.01**^a^	0.92 (0.88–0.96)	** < 0.01** ^a^	0.88 (0.80–0.97)	**0.01**	0.94 (0.89–0.99)	**0.03**
**Patient origin**								
Elective operation	1		1		1		1	
Emergency operation	1.73 (0.90–3.32)	0.10^a^	2.36 (1.67–3.33)	** < 0.01** ^a^	0.99 (0.42–2.35)	0.99	1.65 (1.04–2.60)	**0.03**
**Operation method**								
Open surgery	1.65 (0.59–3.05)	0.11^a^	1.39 (1.01–1.92)	0.046^a^	0.79 (0.35–1.79)	0.57	1.14 (0.75–1.74)	0.53
Minimal invasive surgery	1		1		1		1	
CEA (for every 1 additional in CEA)	0.999 (0.99–1.01)	0.74	1.01 (1.00–1.01)	** < 0.01** ^a^			1.002 (0.99–1.01)	0.23
**Lymphovascular invasion (LVI)**								
LVI (−)	1		1		1			
LVI (+)	2.19 (1.20–4.01)	**0.01**^a^	1.20 (0.84–1.70)	0.32^a^	1.70 (0.83–3.48)	0.15		
**Perineural invasion (PNI)**								
PNI (−)	1		1		1		1	
PNI (+)	1.99 (1.08–3.66)	**0.03**^a^	2.15 (1.53–3.02)	** < 0.01** ^a^	1.11 (0.54–2.27)	0.78	1.75 (1.14–2.67)	**0.01**
**Surgical margin**								
R0 resection	1		1		1			
R1 resection	6.35 (2.49–16.21)	** < 0.01** ^a^	1.16 (0.57–2.37)	0.68	20.36 (5.96–69.61)	** < 0.01**		
**Tumor grade**								
Grade 1–2	1		1				1	
Grade 3–4	0.79 (0.25–2.57)	0.70	1.67 (0.88–3.18)	0.12^a^			0.73 (0.28–1.88)	0.51
**T stage**								
T1			1	** < 0.01** ^a^			1	** < 0.01**
T2			1.12 (0.10–12.33)				1.18 (0.11–13.03)	
T3	1		6.50 (0.90–46.69)		1		3.53 (0.48–26.03)	
T4	2.85 (1.52–5.36)	** < 0.01** ^a^	15.56 (2.16–112.2)		1.68 (0.76–3.73)	0.20	6.59 (0.87–49.72)	
**N stage**								
N0								
N1			1				1	
N2			2.18 (1.59–3.02)	** < 0.01** ^a^			2.00 (1.36–2.94)	** < 0.01**
Tumor size (for every additional 1 cm)	0.97 (0.85–1.10)	0.61	1.08 (1.00–1.16)	0.06^a^			1.02 (0.92–1.13)	0.75
Lymph node harvested (for every additional 1 lymph node harvested)	0.98 (0.95–1.01)	0.20	0.996 (0.98–1.01)	0.55				
Adjuvant chemotherapy								
No	1		1		1		1	
Yes	2.28 (1.20–4.33)	**0.01** ^a^	0.74 (0.51–1.08)	0.12 ^a^	3.38 (1.49–7.66)	** < 0.01**	0.98 (0.58–1.68)	0.95

Significant values are in bold.

*BMI* Body mass index, *CEA* carcinoembryonic antigen.

^a^Only variables with p < 0.2 in univariate analysis were included to multivariate analysis.

In multivariate analysis, RCC (HR 2.35, CI 1.14–4.85, *p* = 0.02), R1 resection (HR 20.36, CI 5.96–69.61, *p* < 0.01), and adjuvant chemotherapy (HR 3.38, CI 1.49–7.66, *p* < 0.01) were risk factors for shorter TTR in stage II colon cancer. On the other hand, emergent operation (HR 1.65, CI 1.04–2.60, *p* = 0.03), perineurial invasion (HR 1.75, CI 1.14–2.67, *p* = 0.01), advanced T stage (T2 vs T1 HR 1.18, CI 0.11–13.03; T3 vs T1 HR 3.53 CI 0.48–26.03; T4 vs T1 HR 6.59 CI 0.87–49.72, *p* < 0.01) and N_2_ stage (HR 2.00, CI 1.36–2.94, *p* < 0.01) were risk factors for shorter TTR in stage III colon cancer. BMI was associated with lower risk of recurrence in both stage II (HR 0.88, CI 0.80–0.97, *p* = 0.02) and stage III (HR 0.94, CI 0.89–0.99, *p* = 0.03) colon cancer.

### Propensity score matching

**Figure 5:**
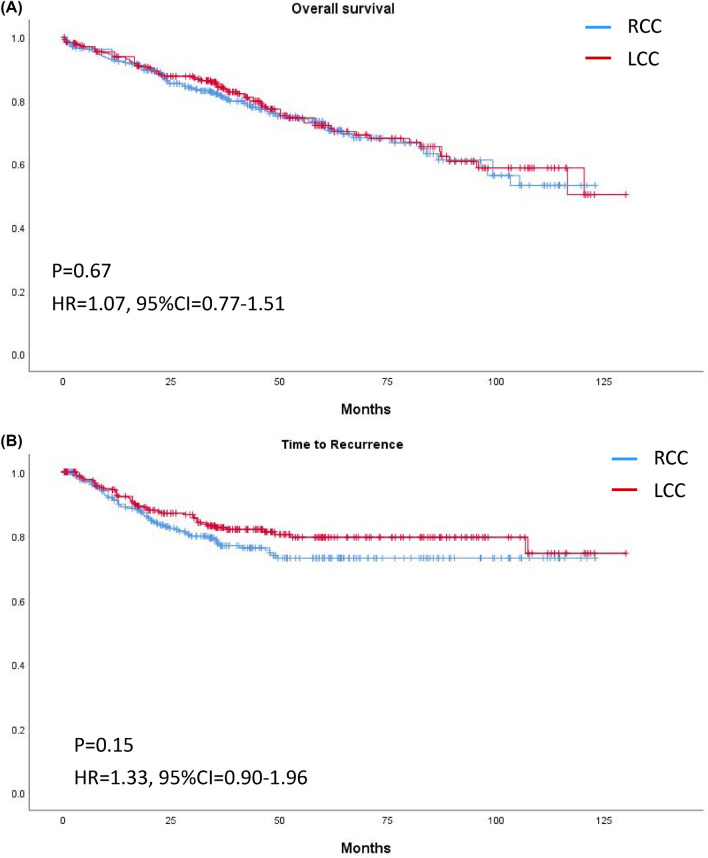
Kaplan–Meier curves of (**A**) overall survival and (**B**) time to recurrence of right-sided and left-sided colon cancer of any stage after PSM.

**Figure 6:**
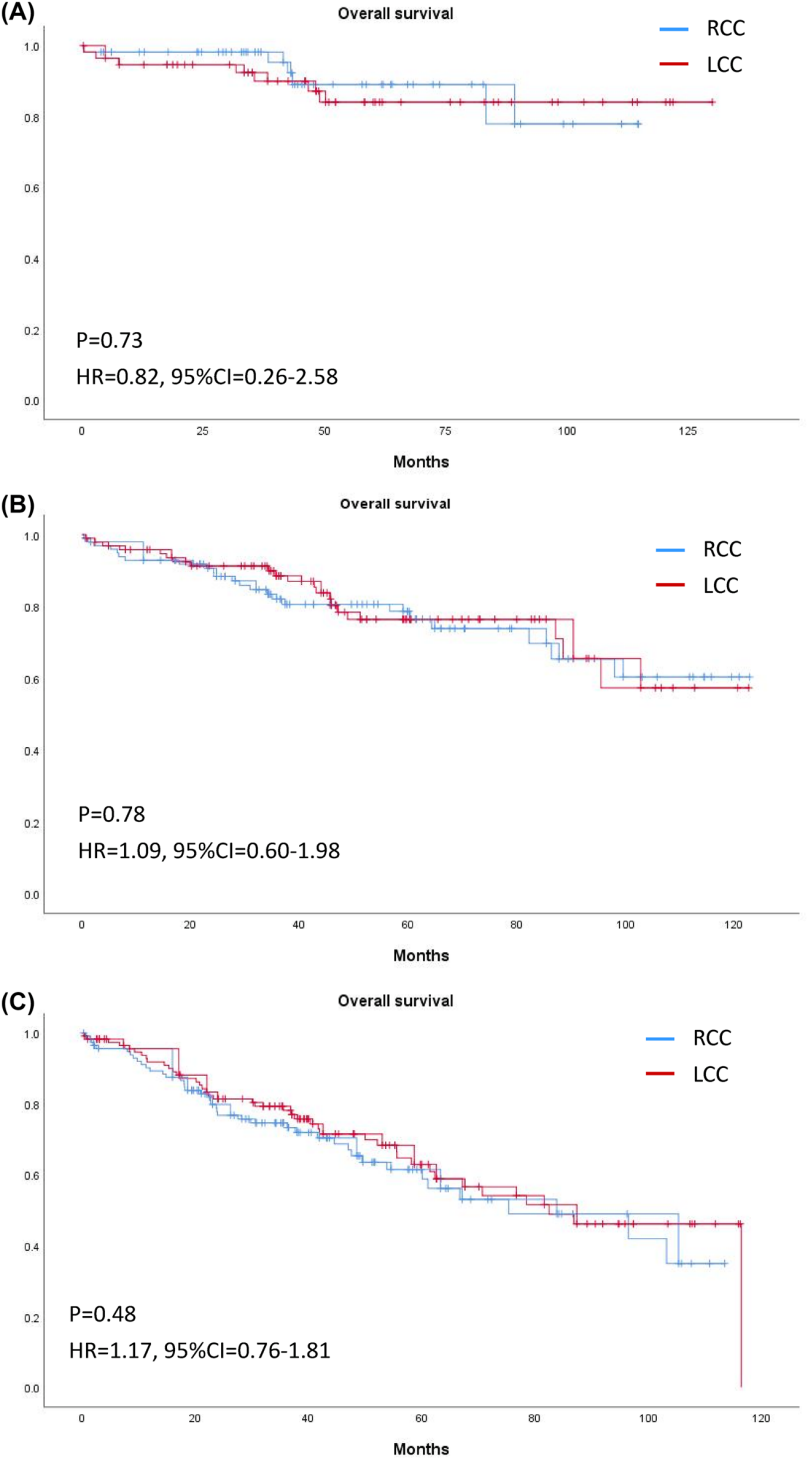
Kaplan–Meier curves of overall survival of right-sided and left-sided colon cancer in (**A**) Stage I, (**B**) Stage II, and (**C**) Stage III after PSM.

**Figure 7:**
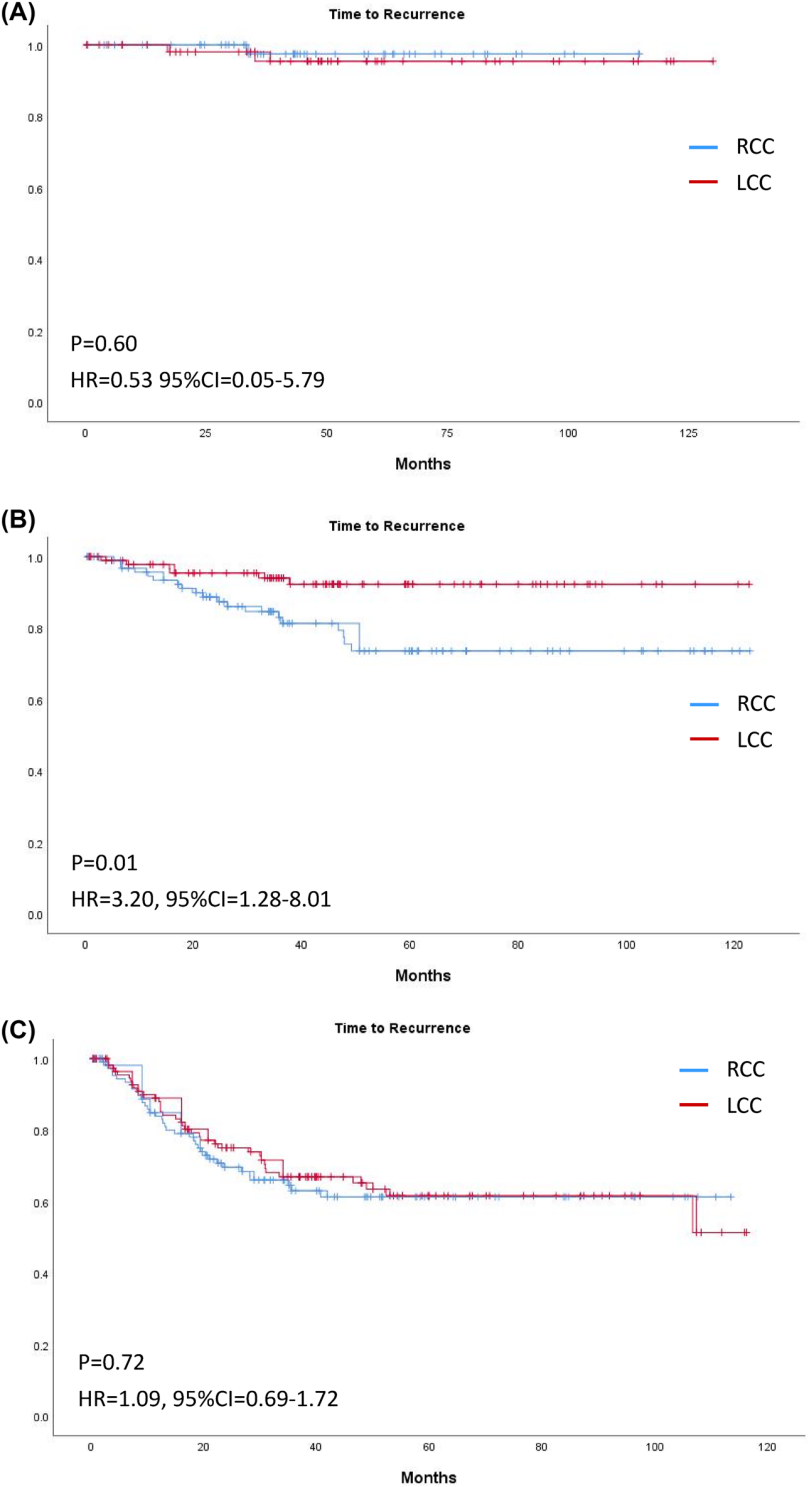
Kaplan–Meier curves of time to recurrence of right-sided and left-sided colon cancer in (**A**) Stage I, (**B**) Stage II, and (**C**) Stage III after PSM.

After PSM, 271 pairs of patients were successfully matched and were listed in Table 4. All variables showed no significance except of number of lymph node harvested (26 (20–36) vs. 22 (17–29), *p* < 0.01). Figure 5, 6, 7 demonstrated survival curves of OS and TTR between RCC and LCC in all stage and stage stratified. Significant difference was found in TTR of stage II colon cancer patients between RCC and LCC (Fig. 7B, HR 3.20, CI 1.28–8.01, *p* = 0.01). Table 5 demonstrated recurrent risks by univariate and multivariate analysis after PSM. The results of multivariate analysis were mostly compatible with results before PSM, except emergent operation did not show increased risk for recurrence in stage III colon cancer patients (HR 1.37, CI 0.72–2.61, *p* = 0.34).

**Table 4:** Baseline characteristics after propensity score matching.

Characteristic	Right-sided cancer (N = 271)	Left-sided cancer (N = 271)	*P* value
Age (years)	66(58–76)	65 (59–76)	0.67
Sex, Male, N (%)	129 (47.6%)	129 (47.6%)	1
Body mass index	24.1 ± 4.1	24.1 ± 4.0	0.96
**Patient origin, N (%)**			0.66
Elective operation	246 (90.8%)	243 (89.7%)	
Emergency operation	25 (9.2%)	28 (10.3%)	
**Operation method, N (%)**			0.48
Open surgery	160 (59.0%)	168 (62.0%)	
Minimal invasive surgery	111 (41.0%)	103 (38.0%)	
CEA	3.5 (1.9–9.6)	3.8 (1.9–8.4)	0.68
Tumor size (cm)	4.5 (3.0–6.1)	4.3 (3.0–6.0)	0.41
Lymphovascular invasion, N (%)	128 (47.2%)	132 (48.7%)	0.73
Perineural invasion, N (%)	106 (39.1%)	107 (39.5%)	0.93
**Tumor grade, N (%)**			0.21
Grade 1–2	251 (92.6%)	258 (95.2%)	
Grade 3–4	20 (7.4%)	13 (4.8%)	
**Location**			
Cecum, N (%)	42		
Ascending colon, N (%)	129		
Hepatic flexure, N (%)	33		
Transverse colon, N (%)	67		
Splenic flexure, N (%)		17	
Descending colon, N (%)		14	
Descending-sigmoid junction, N (%)		16	
Sigmoid colon, N (%)		224	
**T stage, N (%)**			
T1	32 (11.8%)	31 (11.4%)	0.84
T2	36 (13.3%)	39 (14.4%)	
T3	143 (52.8%)	149 (55.0%)	
T4	60 (22.1%)	52 (19.2%)	
**N stage, N (%)**			
N0	154 (56.8%)	153 (56.5%)	0.99
N1	72 (26.6%)	72 (26.6%)	
N2	45 (16.6%)	46 (17.0%)	
**AJCC stage, N (%)**			
I	55 (20.3%)	57 (21.0%)	0.96
II	99 (36.5%)	96 (35.4%)	
III	117 (43.2%)	118 (43.5%)	
No. of lymph node harvested	26 (20–36)	22 (17–29)	** < 0.01**
Adjuvant chemotherapy, N (%)	136 (50.2%)	133 (49.1%)	0.80
K-ras mutation, no. of positive/no. of test (%)	68/149 (45.6%)	37/116 (31.9%)	**0.02**
dMMR, no. of positive/no. of test (%)	17/42 (40.5%)	5/27 (18.5%)	0.06
MSI-H, no. of positive/no. of test (%)	5/16 (31.3%)	1/11 (9.1%)	0.17
R1 resection, N (%)	13 (4.8%)	10 (3.7%)	0.52
Follow time (month)	38.5 (23.7–62.6)	43.1 (24.0–65.7)	0.32

Significant values are in bold.

*CEA* carcinoembryonic antigen, *dMMR* Deficient mismatch repair, *MSI-H* microsatellite instability-high.

**Table 5:** Univariate and multivariate analysis^a^ for TTR by Cox Proportional Hazards Regression in stage II and stage III colon cancer after PSM.

Characteristic	Unadjusted analysis				Adjusted analysis			
	Stage II		Stage III		Stage II		Stage III	
	HR (95%CI)	*P* value	HR (95%CI)	*P* value	HR (95%CI)	*P* value	HR (95%CI)	*P* value
**Location **								
Right	3.20 (1.28–4.48)	**< 0.01**^a^	1.09 (0.69–1.72)	0.72	4.26 (1.61–11.28)	**< 0.01**		
Left	1		1		1			
Age (for every 1 additional year in age)	0.99 (0.96–1.02)	0.42	1.01 (0.99–1.02)	0.55				
**Gender**								
Male	0.74 (0.33–1.64)	0.45	0.997 (0.63–1.57)	0.99				
Female	1		1				1	
BMI (for every 1 additional in BMI)	0.90 (0.81–1.00)	**0.049**^a^	0.93 (0.87–0.99)	**0.02**^a^	0.87 (0.77–0.98)	**0.02**	0.91 (0.85–0.97)	**<0.01**
**Patient origin**								
Elective operation	1		1		1		1	
Emergency operation	2.57 (0.88–7.50)	0.08^a^	1.84 (1.01–3.35)	**0.04**^a^	1.12 (0.34–3.70)	0.85	1.37 (0.72–2.61)	0.34
**Operation method**								
Open surgery	1.75 (0.80–3.83)	0.16^a^	1.45 (0.92–2.29)	0.11^a^	0.95 (0.38–2.38)	0.91	1.21 (0.73–20.1)	0.47
Minimal invasive surgery	1		1		1			
CEA (for every 1 additional in CEA)	0.997 (0.97–1.03)	0.78	1.00 (0.997–1.01)	0.37				
**Lymphovascular invasion (LVI)**								
LVI (−)	1		1		1			
LVI(+)	1.91 (0.88–4.19)	0.11^a^	1.21 (0.72–2.03)	0.48	1.83 (0.78–4.31)	0.17		
Perineural invasion (PNI)								
PNI (−)	1		1		1		1	
PNI (+)	1.71 (0.78–3.78)	0.18^a^	2.01 (1.22–3.31)	**< 0.01**^a^	1.60 (0.70–3.65)	0.27	1.79 (1.04–3.06)	**0.04**
**Surgical margin**								
R0 resection	1		1		1			
R1 resection	14.80 (4.87–45.01)	**< 0.01**^a^	1.01 (0.41–2.49)	0.99	25.06 (5.59–112.46)	**< 0.01**		
**Tumor grade**								
Grade 1–2	1		1					
Grade 3–4	0.44 (0.06–3.24)	0.42	0.99 (0.36–2.71)	0.98				
**T stage**								
T1			1	**< 0.01**^a^			1	**<0.01**
T2			0.64 (0.04–10.30)				0.84 (0.05–13.52)	
T3	1		3.43 (0.47–24.99)		1		2.85 (0.38–21.332)	
T4	3.79 (1.70–8.44)	**< 0.01**^a^	7.42 (1.01–54.32)		2.04 (0.81–5.14)	0.13	5.82 (0.77–44.28)	
**N stage**								
N0								
N1			1				1	
N2			2.01 (1.27–3.17)	**< 0.01**^a^			1.74 (1.09–2.77)	**0.02**
Tumor size (for every additional 1 cm)	0.94 (0.79–1.12)	0.49	1.07 (0.97–1.18)	0.195^a^			1.01 (0.90–1.13)	0.83
Lymph node harvested (for every additional 1 lymph node harvested)	0.99 (0.96–1.03)	0.74	0.997 (0.98–1.02)	0.72				
**Adjuvant chemotherapy**								
No	1		1		1			
Yes	2.37 (0.99–5.68)	0.053^a^	1.05 (0.59–1.84)	0.88	2.69 (1.06–6.83)	**0.04**		

Significant values are in bold.

*BMI* Body mass index, *CEA* carcinoembryonic antigen.

^a^Only variables with p<0.2 in univariate analysis were included to multivariate analysis.

## Discussion

This study was a large-scale retrospective study. We followed more than 1000 patients who underwent curative resection for stage I, II, and III colon cancer, with the longest follow-up time more than 10 years. This study demonstrated that the sidedness of colon cancer did not influence OS but influenced TTR in stage II colon cancer. We also observed that RCC, lower BMI, R1 resection and chemotherapy were risk factors for shorter TTR in stage II colon cancer, whereas lower BMI, perineurial invasion, advanced T and N stage were risk factors for shorter TTR in stage III colon cancer. To compensate for the possible bias in this retrospective study, we performed propensity score matching. The results of multivariate analysis were mostly compatible with the results before PSM, which makes this study more reliable.

In previous population-based studies, the OS rate was highly different between stage I, II, and III RCC and LCC. A study of 91,416 patients demonstrated that OS in stage I and II RCC was longer than in stage I and II LCC^14^. In a study of 77,978 patients, Meguid et al. reported that OS in stage II and III RCC was worse than in stage II and III LCC^6^. However, in a study of 53,801 patients over 65 years of age, Weiss et al. reported that RCC had longer OS than LCC in stage II but worse OS in stage III^16^. The age at diagnosis of RCC is approximately 3.0 to 4.6 years older than that for LCC^6,17,18^, and only one-third of patients with stage I–III colon cancer die from the cancer^19^. Therefore, OS does not truly reflect the difference in prognosis between RCC and LCC^20^; hence, we used TTR to compare the risk factors of RCC and LCC.

This study also noted differences between RCC and LCC and reinforced the consensus that RCC and LCC can be regarded as distinct cancers. However, our results contradicted previous studies regarding their specific differences. First, many previous population-based studies have reported a higher proportion of women with RCC than LCC^6,14,16^; this was also true in our study. However, in our results, women only composed a higher proportion of patients with stage I and II RCC, not stage III. Second, several studies have noted that RCC has a larger tumor size and presents as a later stage than LCC^6,21^. In our study, tumor size was significantly larger in stage III RCC than in stage III LCC but not in stage II RCC. It is generally believed that LCC tends to manifest in symptoms such as changes in bowel habits and bleeding earlier than RCC, and consequently, LCC is diagnosed earlier than RCC^22^. However, the results of this study indicate that the consensus that LCC is often detected at an earlier stage than RCC may need to be reevaluated. A relatively small tumor size with lymph node metastasis may be the typical representation of LCC at the time of diagnosis. Third, lymphovascular invasion and tumor grade are powerful prognostic factors in colon cancer^23^, and many studies have reported that RCC has a higher rate of lymphovascular invasion and a higher tumor grade than LCC^12,20,21,24^. However, we observed that tumor grade and the incidence of lymphovascular invasion are significantly higher in stage II RCC than in stage II LCC but not in stage III RCC. The high rate of lymphovascular invasion and the presence of grade 3–4 tumor cells shortened TTR in stage II RCC compared with that for stage II LCC. In our study, although stage III RCC had a larger tumor size and more advanced T stage than stage III LCC, cancer recurrence did not differ if patients underwent appropriate radical resection and adjuvant chemotherapy; moreover, we observed no difference in tumor size between stage II RCC and LCC. However, RCC had a shorter TTR because of a higher proportion of lymphovascular invasion and tumor grade. Therefore, we believe that stage II RCC can be regarded as a unique category of colorectal cancer, and postoperative treatment and follow-up strategies need to be accordingly tailored.

Several studies have also suggested that colon cancer heterogeneity is different between RCC and LCC. Microsatellite instability (MSI) and dMMR are more common in RCC. Patients with stage II colon cancer and MSI or dMMR tumors had a better prognosis in RCC than LCC, but in patients with stage III colon cancer, MSI or dMMR provided no benefit to prognosis^12,25,26^. A recent study reported that in patients with dMMR stage II colon cancer, 5-FU based chemotherapy did not improve survival and could even worsen prognosis^27^. The National Comprehensive Cancer Network Clinical Practice Guidelines in Oncology (2017) recommended that MMR status be checked in stage II colon cancer^3^. Our institute follows this treatment guideline and has tested MMR status for colon cancer since 2017. According to the limited MMR test data, nearly half of patients with RCC had dMMR (45.5%), which is 10–20% higher than in previous studies^12,28^. This also explains why chemotherapy was a risk factor for stage II RCC because many patients underwent adjuvant chemotherapy without an MMR status test before 2017.

In previous studies, no differences in BMI were reported between patients with RCC and LCC^23,24,29^. In this study, however, patients with RCC and stage III RCC had significantly lower BMI than did patients with LCC and stage III LCC, respectively. This may be because patients with RCC are predominantly female and older, which are two groups that generally have a lower BMI. We observed increased BMI to be a positive prognostic factor for patients with both stage II and stage III cancer. By contrast, previous studies have reported obesity or a BMI of > 30 kg/m^2^ to be risk factors for cancer recurrence and cancer-related mortality in colorectal cancer^30–33^. In agreement with our study, Sinicrope et al. reported that overweight patients (BMI 25–29.9 kg/m^2^) had better OS in stage II and III colon cancer^34^; the authors speculated that relatively healthy patients in this BMI range can tolerate operations and adjuvant chemotherapy^34^. We speculate that patients with a low BMI have too little visceral fat to cover the tumor, and thus, the tumor can easily invade the surrounding organs or spread. Patients with obesity usually have comorbid diseases, and complications often occur during surgery or chemotherapy. Most patients (91%) in this study had a BMI below 30, and we observed that increased BMI was associated with longer TTR.

This study had several limitations. First, this was a retrospective study at a single institution, which makes our study susceptible to bias and confounding factors despite the large study population. Second, only 33 patients (0.3%), 110 patients (11%) and 488 patients (48%) had an MSI status, MMR status or K-RAS mutation test, respectively. Because of the low number of patients with an MSI status, MMR status or K-RAS mutation test, we did not analyze the effect of these variables on prognosis in this study. Third, detailed chemotherapy data were not available in this study. Patients who received oral chemotherapy, intravenous chemotherapy, and incomplete chemotherapy courses were all classified as having received adjuvant chemotherapy. This may underestimate the effects of chemotherapy. Finally, complete mesocolic excision (CME) in RCC increases OS and DFS, and cancer stage may be underdiagnosed in patients without CME^35–37^. CME is not routinely performed for RCC at our institute, and CME status is not always recorded in medical records, thus affecting the analysis of prognosis.

In conclusion, sidedness is an independent risk factor for cancer recurrence in stage II colon cancer; patients with stage II RCC had shorter TTR than did those with stage II LCC. Furthermore, stage III RCC had a more advanced T stage, but this did not influence TTR in stage III colon cancer. Further research is needed to evaluate the differences of sidedness by using clinicopathological and genetic factors.

## Data Availability

The data generated in this study are available on request from the corresponding author.
